# Alterations of brain activity in patients with alcohol use disorder: a resting-state fMRI study

**DOI:** 10.1186/s12888-023-05361-z

**Published:** 2023-11-30

**Authors:** Xia Ruan, Zhiyan Song, Jie Zhang, Tingting Yu, Jun Chen, Tiantian Zhou

**Affiliations:** 1 Department of Radiology, Renmin Hospital of Wuhan University, Wuhan, Hubei Province 430060 People’s Republic of China; 2 Department of Radiology, Wuhan No.1 Hospital, Wuhan, Hubei Province 430030 People’s Republic of China; 3 Department of Radiology, Hubei Cancer Hospital, Tongji Medical College, Huazhong University of Science and Technology, Wuhan, Hubei Province 430079 People’s Republic of China; 4 Department of Medical Imaging, Wuhan Pulmonary Hospital, Wuhan, Hubei Province 430030 People’s Republic of China

**Keywords:** Alcohol use disorder, Degree centrality, Functional connectivity, Resting-state functional magnetic resonance imaging, Brain activity

## Abstract

**Background:**

Alcohol use disorder (AUD) has a negative impact on one’s health and wastes a lot of societal resources since it damages one’s brain tissue. Yet the knowledge of the neural mechanisms underlying alcohol addiction still remains limited. This study aims to investigate the neural mechanisms underlying alcohol addiction by using voxel-wise binarized degree centrality (DC), weighted DC and functional connectivity (FC) methods to analyze brain network activity in individuals with AUD.

**Methods:**

Thirty-three AUD patients and 29 healthy controls (HC) participated in this study. Binarized and weighted DC approach coupled with a second seed-based FC algorithm was used to assess the abnormal intrinsic hub features in AUD. We also examined the correlation between changes in functional network nodes and the severity of alcohol dependence.

**Results:**

Thirty AUD patients and 26 HC were retained after head motion correction. The spatial distribution maps of the binarized DC and weighted DC for the AUD and HC groups were roughly similar. In comparison to HC, the AUD group had decreased binarized DC and decreased weighted DC in the left precentral gyrus (PreCG) and the left inferior parietal lobule (IPL). Significantly different brain regions in the DC analysis were defined as seed points in the FC analysis. Compared with HC, changes in FC within the right inferior temporal gyrus (ITG), right middle temporal gyrus (MTG), left dorsolateral superior frontal gyrus (SFGdor), bilateral IPL, left precuneus (PCUN), left lingual gyrus (LING), right cerebellum_crus1/ITG/inferior occipital gyrus (IOG) and right superior parietal gyrus (SPG) were observed. The correlation analysis revealed that FC of right MTG-right PreCG was negatively correlated with MAST scores, and FC of right IPL-left IPL was positively correlated with ADS scores.

**Conclusions:**

Alcohol use disorder is associated with aberrant regional activities in multiple brain areas. Binarized DC, weighted DC and FC analyses may be useful biological indicators for the detection of regional brain activities in patients with AUD. Intergroup differences in FC have also been observed in AUD patients, and these variations were connected to the severity of the symptoms. The AUD patients with lower FC value of the right IPL - left IPL has a lighter dependence on alcohol. This difference in symptom severity may be a compensation for cognitive impairment, indicating a difference in pathological pathways. Future AUD research will now have a fresh path thanks to these discoveries.

## Background

Alcohol use disorder (AUD), one of the most prevalent psychiatric disorders in the world, is defined by compulsive drinking behavior and continuous, excessive, uncontrolled alcohol consumption [1]. AUD is closely related to the problems of public health, social, criminal, and mental health [2]. Continued excessive drinking can have an adverse effect on several organs and is also directly related to a number of malignancies [3].

A number of novel biomarkers for the diagnosis of AUD have been identified through brain imaging techniques in recent years. Voxel-based morphometry (VBM) studies revealed that alcohol consumption was significantly associated with lower total and local gray matter volume [4, 5]. Reduced grey matter volume is associated with the duration time of alcohol dependence and lifetime alcohol consumption [6]. Whole-brain voxel-wise analyses of diffusion tensor imaging (DTI) found that AUD patients had extensive damage to white matter (WM) structures [7]. AUD not only causes structural damage but also functional changes in brain regions. In a proton Magnetic Resonance Spectroscopy (MRS) study, alcohol intake was negatively correlated with Choline/Creatine ratio in the left prefrontal cortex, perhaps indicating the severity of alcohol abuse [8]. The left parietal region and the prefrontal area were also less perfused in AUD patients [9]. Prior research by our team revealed that the efficiency of the brain network in the AUD group was significantly lower [10].

The resting-state functional magnetic resonance imaging (rs-fMRI) technique is a non-invasive method that is widely used to quantify the neural network structure and functional brain properties of AUD patients [11]. Binarized and weighted degree centrality (DC) can reflect the density and strength of whole-brain functional connectivity (FC), which has the advantages of avoiding the influence of subjective selection by not defining the seed points, and can indirectly reflect the importance and changes of brain network nodes [12]. Many diseases have been studied using the voxel-wise DC approach, but the use of binarized and weighted DC methods in the study of AUD patients has been limited. This study used rs-fMRI to track DC and FC changes in brain activity in AUD patients and correlate these changes with clinical and neuropsychological information.

## Methods

### Subjects

This protocol was approved by Medical Ethics Committee of Renmin Hospital of Wuhan University, and followed the Helsinki Declaration. The consent of all subjects was obtained in writing.

Thirty-three right-handed male AUD patients and 29 age-, sex-, handedness-, and education-matched healthy controls (HC) were recruited from the primary-care outpatient department of Renmin Hospital of Wuhan University. Before the MRI scan, the psychiatrist assessed their neurological and physical capacities as well as their medical histories. If any of the following conditions were present, patients were excluded: (1) an individual who exhibits psychotic symptoms or whose first-degree relative has been diagnosed with psychosis; (2) history of addiction to substances other than alcohol; (3) with organic brain disease or severe physical disease; (4) a history of cranial trauma, cranial surgery, brain tumor and coma; (5) people with previous seizures or a family history of epilepsy; (6) patients who have been treated with antipsychotic medication or who are receiving medication; (7) claustrophobia or any MRI contraindications.

Inclusion criteria of AUD patients: all AUD patients have been drinking alcohol for no less than 10 years; AUD patients met diagnostic criteria for AUD in the Fifth Edition of the American Diagnostic and Statistical Manual of Mental Disorders (DSM-V) (the ICD-10-CM code: F10.10/F10.20) [13]; Alcohol drinking scale (ADS) score ≥ 14 and Michigan Alcoholism Screening Test (MAST) score ≥ 6 [14].

HCs were defined as people who had never or rarely consumed alcohol (< 1 standard unit per time) [15].

The time interval between the last alcohol consumption and MR examination was three weeks for all subjects in this study to exclude the effects of acute alcohol intake.

### Cognitive and alcohol level assessment

The Mini-Mental State Examination (MMSE) and Montreal Cognitive Assessment (MoCA) were completed by all subjects before MRI scanning; we only collected ADS and MAST scores from the AUD group.

### MRI acquisition

MRI images were acquired using a 3.0 Tesla MRI scanner (Discovery 750 W, GE Healthcare, Milwaukee, WI, USA). To avoid subject head movement artifacts during the experiment, the matching rubber soft plugs were used to make the head fixed. We also used soft foam ear plugs to reduce equipment noise. All subjects were asked to be quiet and relaxed, remain awake, keep their eyes closed and think of nothing in particular. Participants with lesions of brain were exclude from T2WI and T2-FLAIR images. The scanning parameters of fMRI data are as follows: repetition time (TR) = 2000 ms; echo time (TE) = 25 ms; field of view (FOV) = 240 mm×240 mm; flip angle (FA) = 90°; slice thickness = 3.5 mm; no slice gap; matrix size = 64 × 64; and slice number = 40. The 3D T1-weighted structural images were obtained in sagittal position: TR/TE = 8.5/3.3; FOV = 240 mm×240 mm; FA = 12°; slice thickness = 1.0 mm; no slice gap; matrix size = 256 × 256; slice number = 156; and voxel size = 1 mm×1 mm×1 mm.

### Image processing and analysis

#### Data preprocessing

Rs-fMRI data were preprocessed by Statistical Parametric Mapping software (SPM12) (http://www.fil.ion.ucl.ac.uk/spm) and Resting-State fMRI Data Analysis Toolkit plus (RESTplus) (http://restfmri.net/forum/RESTplus). The main preprocessing steps were as follows: (1) remove the first 10 time points because of inhomogeneities in the magnetic field and subjects’ maladaptive interferences with the environment, and the last 230 time points of each data were finally retained for subsequent analysis; (2) slice timing; (3) realign, subjects with head movement > 2.0 mm or 2.0° were excluded; (4) normalizing the data to the Montreal Neurological Institute (MNI) space by the technique of DARTEL [16]; (5) detrend; (6) nuisance covariates regression, Friston-24 parameters were applied, white matter and cerebrospinal fluid signals were removed; (7) filter (0.01–0.08 Hz).

#### Degree centrality analysis

For DC calculation, we analyzed preprocessed fMRI data using the RESTplus software (http://restfmri.net/forum/RESTplus). DC maps of the entire brain were created by calculating the correlation between the time series of each voxel and that of all other voxels. A whole gray matter functional connectivity matrix was generated for each participant. A threshold value of r > 0.25 was selected to screen out correlation values [12, 17]. Fisher’s r-to-z transformation was applied to obtain DC z-score maps for each subject. The resulting maps were smoothed using a 6 mm × 6 mm × 6 mm Gaussian kernel, which helps reduce noise and enhance spatial consistency. Finally, binarized DC maps and weighted DC maps were obtained for each individual. Binarized DC represents the number of positive functional connections between a voxel and all other voxels within the gray matter, while weighted DC sums the coefficients of positive functional connectivity for each voxel.

#### Functional connectivity analysis

To smooth the preprocessed data, we applied a Gaussian kernel with 6 mm × 6 mm × 6 mm. The DC results’ peak points were predetermined at the seed positions. All seed regions had a 6 mm radius. Then, we performed an FC analysis between the seed region and all other brain voxels. Fisher’s r-to-z transformation was then utilized to produce zFC maps for each participant.

### Statistical analysis

#### Demographic and clinical data

Baseline clinical information was compared between the two groups using SPSS 26.0. Data for continuous variables were expressed as mean ± standard deviation. Demographic and clinical data for both groups were tested by the Shapiro-wilk test. For data with non-normal distribution, Mann-Whitney U test was employed, while data with normal distribution, independent samples t-test was used for group comparison. We set the threshold at a significant level of *P* < 0.05.

#### Degree centrality and functional connectivity

To compare spatial distribution maps of binarized and weighted DC values within groups, we used the one-sample t-test (*P* < 0.05). We performed the two-sample t-test to analyze the differences of binarized, weighted DC and FC values between the two groups. We used the clustering level family wise error (voxel *P* < 0.001 uncorrected, cluster *P* < 0.05 FWE-corrected) method for multiple comparison correction of the DC results. The corrected cluster size threshold for binarized DC and weighted DC were 77 and 94 voxels, respectively. The statistical and correction methods of the FC analysis were consistent with DC.

#### Correlation analysis

The Pearson’s correlation between the altered DC/FC values and MAST/ADS scores was calculated to examine the relationship between the altered DC/FC values and scores on clinical measures.

## Results

### Demographic and clinical data

Six subjects (3 patients with AUD and 3 HC) were excluded following correction for head motion. Finally, 30 AUD patients and 26 HC were enrolled in this study. The demographic and clinical information of the two groups were presented in Table 1. In terms of gender, age, education, and handedness, there was no significant difference between the two groups. However, the AUD group had lower MMSE and MoCA scores than the HC group (*P* < 0.05).

**Table 1:** Demographic and clinical characteristics

Characteristics	AUD(n = 30)	HC(n = 26)	P-value
Age(years)	53.03 ± 5.49	49.96 ± 6.08	0.052
Gender(male/female)	30/0	26/0	-
Education level(years)	11.17 ± 2.78	10.35 ± 3.07	0.299
Handedness (R/L)	30/0	26/0	-
MoCA	25.17 ± 2.83	26.65 ± 1.98	0.002
MMSE	26.73 ± 1.86	28.19 ± 1.34	0.002
MAST	8.83 ± 3.36	-	-
ADS	17.03 ± 3.54	-	-

Values are expressed as means ± standard deviations or frequencies

*P* < 0.05 was considered statistically significant

AUD, alcohol use disorder; HC, healthy control; MoCA, Montreal Cognitive Assessment; MMSE, Mini-Mental State Examination; MAST, Michigan Alcoholism Screening Test; ADS, Alcohol Drinking Scale

### Degree centrality analysis

**Fig. 1:**
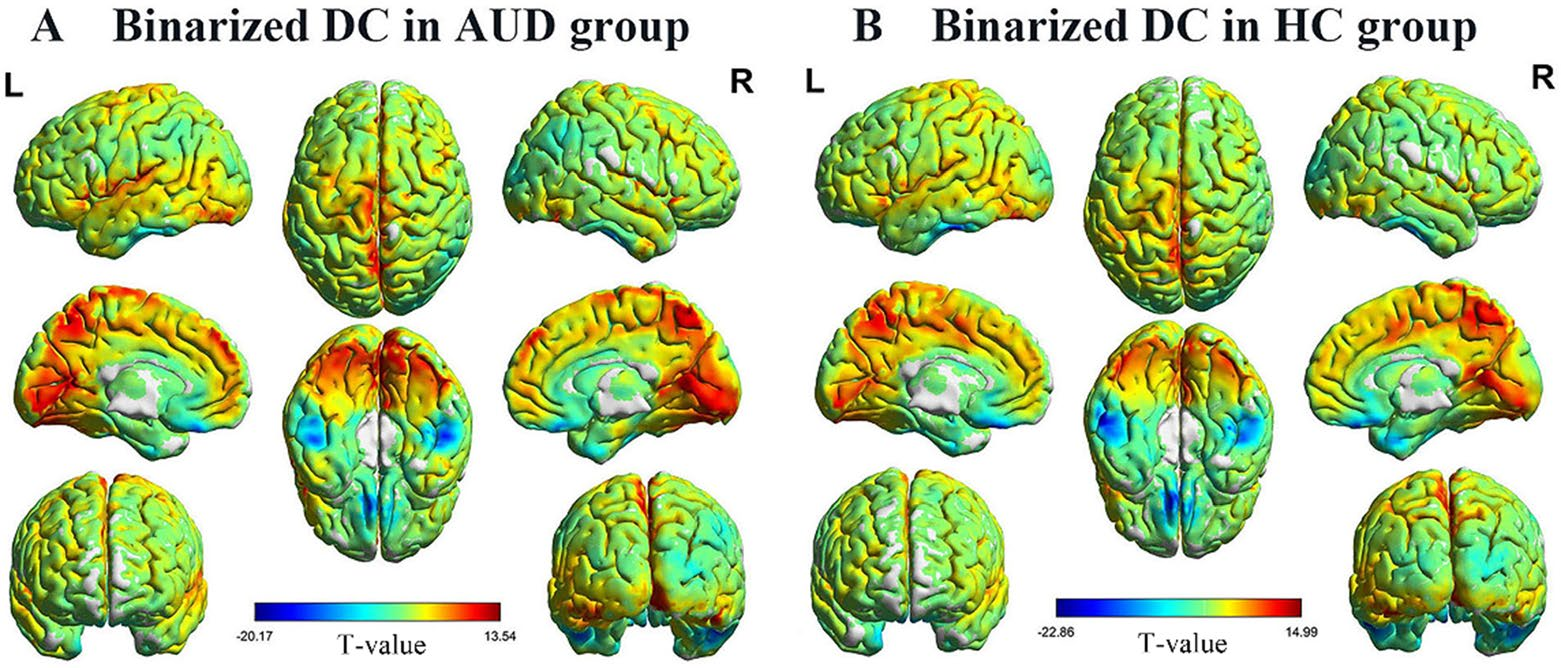
Spatial distribution maps of binarized DC. The spatial distribution of binary DC in the AUD group (**A**) and the HC group (**B**), where the warm color represents the area with high binarized DC value and the cold color represents the area with low binarized DC value. DC, degree centrality; AUD, alcohol use disorder; HC, healthy controls; L, left; and R, right

**Fig. 2:**
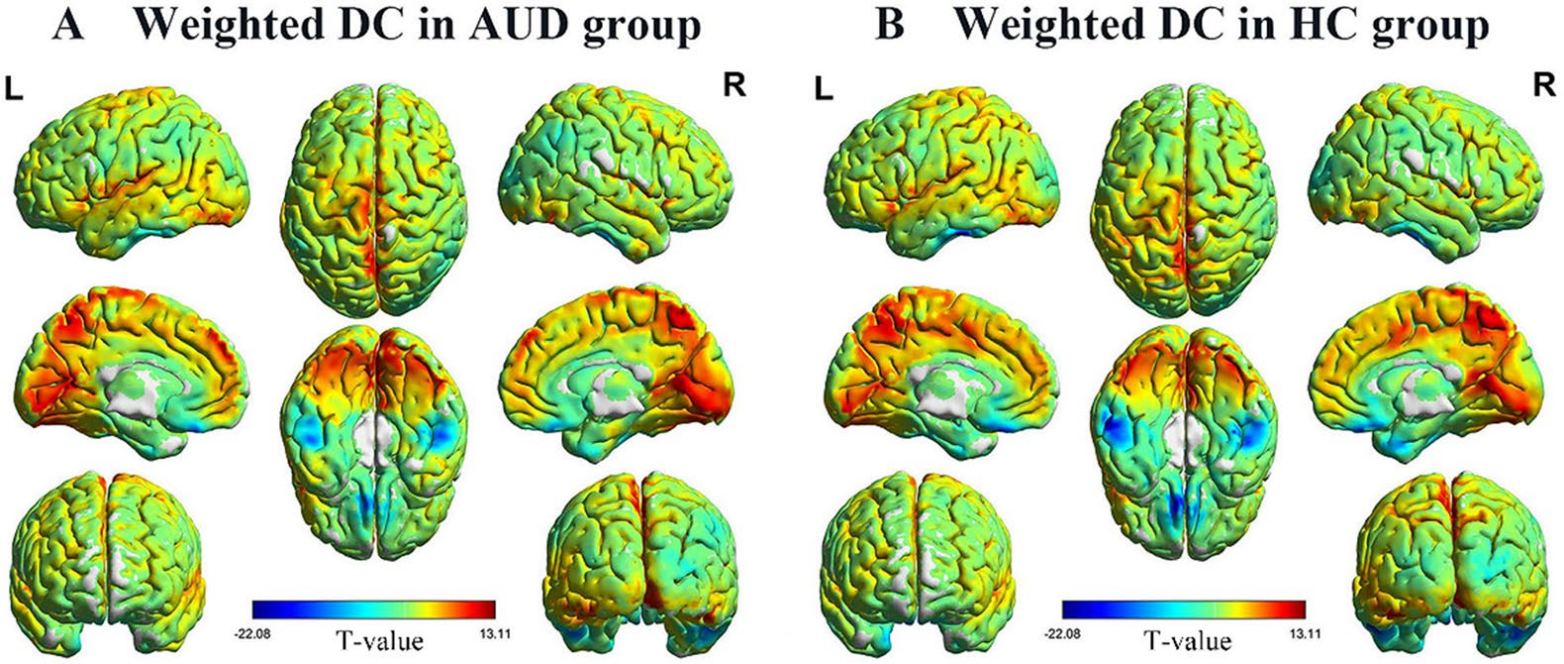
Spatial distribution maps of binarized DC. The spatial distribution of binarized DC in the AUD group (**A**) and the HC group (**B**), where the warm color represents the area with high weighted DC value and the cold color represents the area with low weighted DC value. DC, degree centrality; AUD, alcohol use disorder; HC, healthy controls; L, left; and R, right

One-sample t-test was used for within-group comparisons (*P* < 0.05). The spatial distribution maps of binarized DC values (Fig. 1) and weighted DC values (Fig. 2) in AUD group and control group are broadly similar.

**Fig. 3:**
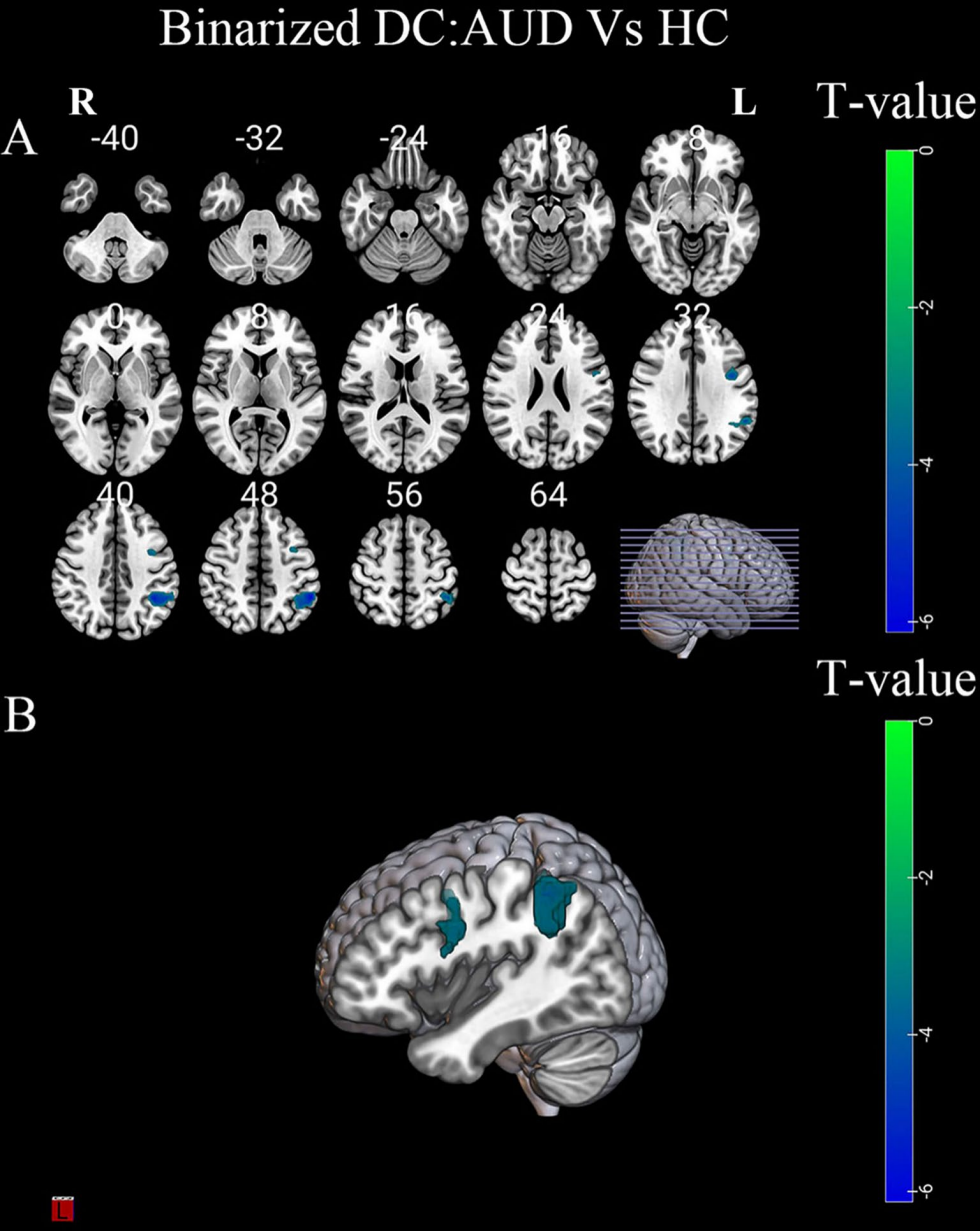
(**A-B**) Differences in binarized DC values between the AUD and HC groups. Green-blue denotes lower DC values in patients with AUD compared to HC. DC, degree centrality; AUD, alcohol use disorder; HC, healthy controls

**Fig. 4:**
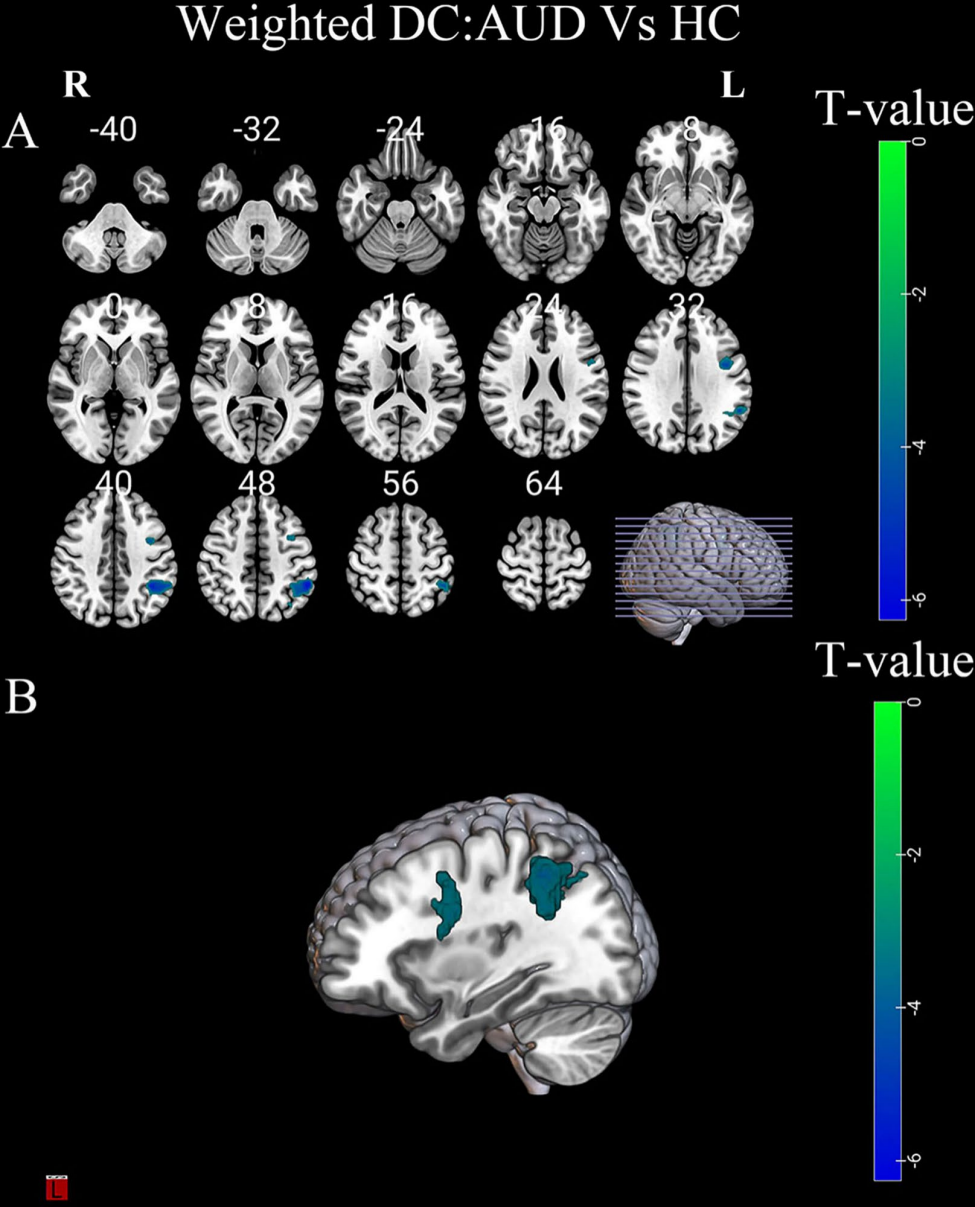
(**A-B**) Differences in weighted DC values between the AUD and HC groups. Green-blue denotes lower DC values in patients with AUD compared to HC. DC, degree centrality; AUD, alcohol use disorder; HC, healthy controls

Compared to HC group, the AUD group had decreased binarized and weighted DC values in the left precentral gyrus (PreCG) and left inferior parietal lobule (IPL) (Table 2; Figs. 3 and 4).

**Table 2:** Brain area differences in degree centrality between patients with AUD and HC (FWE corrected)

Specific classification	Peak MNI coordinate region	Side	Cluster Size	BA	Peak MNI coordinates			T value
					X	Y	Z	
Binarized DC								
	Precentral gyrus	L	77	6	-45	0	30	-5.13
	inferior parietal lobule	L	273	40	-42	-48	36	-6.37
Weighted DC								
	Precentral gyrus	L	94	6	-45	0	30	-4.99
	inferior parietal lobule	L	289	40	-54	-45	51	-6.38

AUD, alcohol use disorder; HC, healthy controls; DC, degree centrality; MNI, Montreal Neurological Institute; BA, Brodmann area; L, left

### Seed-based functional connectivity analysis

**Fig. 5:**
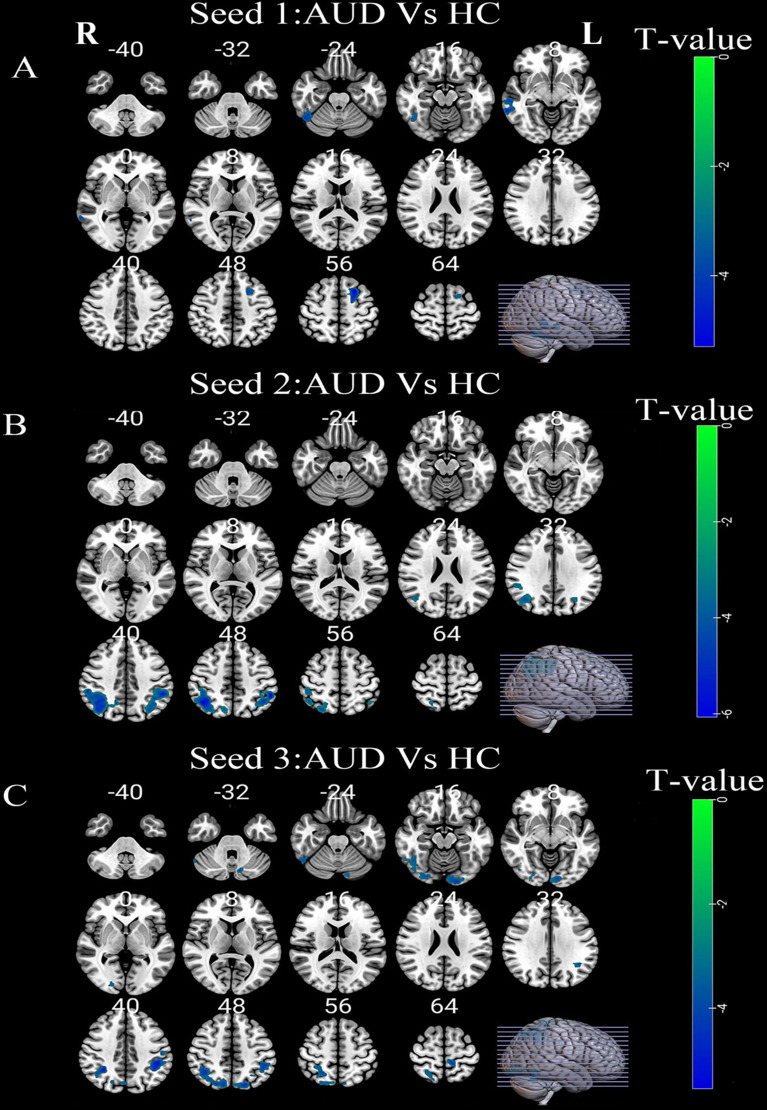
(**A**) Differences in left PreCG-based(-45,0,30) FC between the AUD and HC groups. (**B**) Differences in left IPL-based(-42,-48,36) FC between the AUD and HC groups. (**C**) Differences in left IPL-based(-54,-45,51) FC between the AUD and HC groups. Green-blue denotes lower FC values in patients with AUD compared to HC. PreCG, precentral gyrus; IPL, inferior parietal lobule; FC, functional connectivity; AUD, alcohol use disorder; HC, healthy controls

The left PreCG and left IPL were found to be vitally important in AUD according to DC analysis. The peak point in the DC results was therefore set to the coordinates of the three seeds in subsequent FC analysis. Compared to HC group, the AUD group had a decreased PreCG-based (-45,0,30) FC in right middle temporal gyrus (MTG), right inferior temporal gyrus (ITG), and left dorsolateral superior frontal gyrus (SFGdor), and exhibited significantly lower IPL-based (-42,-48,36) FC in left and right IPL, whereas, a significantly lower IPL-based (-54,-45,51) FC in left lingual gyrus (LING), left precuneus (PCUN), right superior parietal gyrus (SPG), right cerebellum_crus1/ITG/inferior occipital gyrus (IOG), and left IPL was observed (Table 3 and Fig. 5).

**Table 3:** Brain area differences in functional connectivity between patients with AUD and HC (FWE corrected)

Seed area	Peak MNI coordinate region	Side	Cluster Size	BA	Peak MNI coordinates			T value
					X	Y	Z	
Seed 1								
	Inferior temporal gyrus	R	92	-	48	-54	-21	-4.34
	Middle temporal gyrus	R	98	21	66	-45	-3	-4.83
	Dorsolateral superior frontal gyrus	L	147	6	-21	15	57	-5.38
Seed 2								
	Inferior parietal lobule	R	800	40	42	-57	45	-6.12
	Inferior parietal lobule	L	367	40	-54	-45	48	-6.19
Seed 3								
	Precuneus	L	115	3	-12	-39	66	-4.63
	Lingual gyrus	L	163	17	-21	-96	-18	-5.14
	Cerebellum_Crus1/ Inferior temporal gyrus/ Inferior occipital gyrus	R	189	-	54	-57	-30	-4.27
	Superior parietal gyrus	R	457	-	30	-48	39	-5.49
	inferior parietal lobule	L	220	40	-42	-48	36	-5.76

Seed 1, left precentral gyrus (-45,0,30)

Seed 2, left inferior parietal lobule (-42,-48,36)

Seed 3, left inferior parietal lobule (-54,-45,51)

AUD, alcohol use disorder; HC, healthy controls; MNI, Montreal Neurological Institute; BA, Brodmann area; L, left; and R, right

**Fig. 6:**
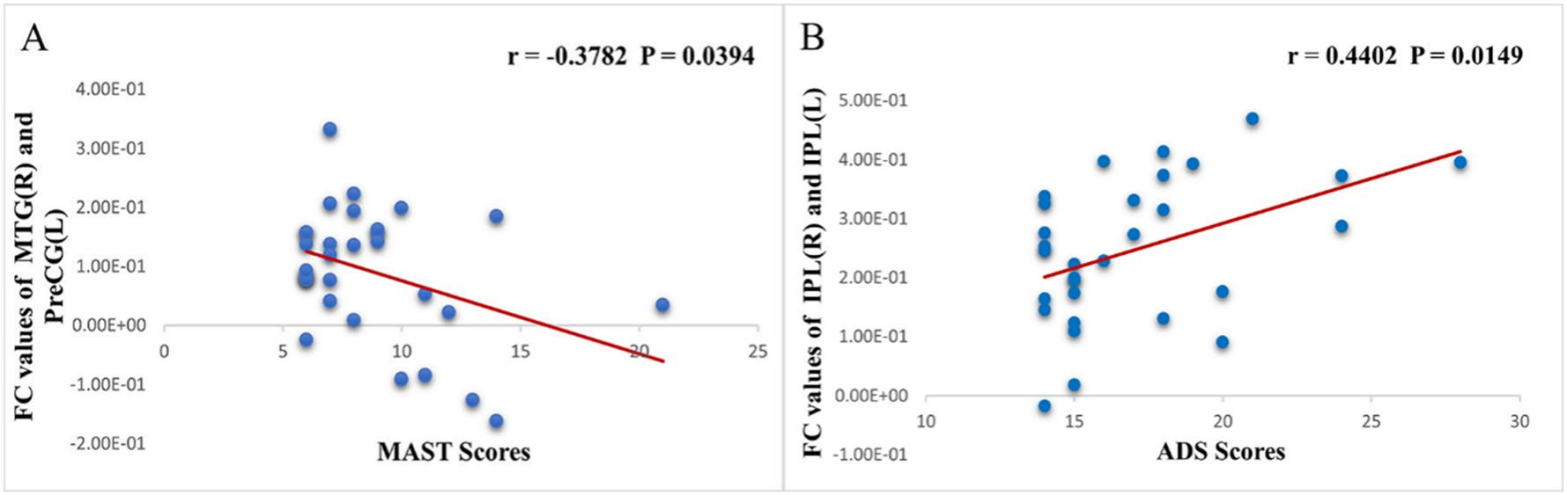
(**A**) Scatter plot showed a negative correlation between MAST scores and the FC values of MTG (R) - PreCG (R) in patients with AUD. (**B**) Scatter plot showed a positive correlation between ADS scores and the FC values of IPL (R) - IPL (L) in patients with AUD

### Correlation analysis

AUD-related measurements and altered FC were shown to be significantly correlated (Table 4; Fig. 6). The FC values of right MTG - right PreCG and that of right IPL - left IPL were correlated with MAST (r = 0.3782, *P* = 0.0394) and ADS (r = 0.4402, *P* = 0.0149), respectively.

**Table 4:** Correlation between AUD and functional connectivity

AUD measures	functional connectivity	r	P
MAST scores	MTG(R) and PreCG(L)	-0.3782	0.0394
ADS scores	IPL(R) and IPL(L)	0.4402	0.0149

Pearson correlation coefficient r representing the strength and direction of linear relationships between an AUD-related measures and connectivity strength between regions in the cluster of regions identified in the FC

MTG, middle temporal gyrus; PreCG, precental gyrus; IPL, inferior parietal lobule; L, left; and R, right

## Discussion

In this study, we investigated alterations in binarized, weighted degree centrality and functional connectivity in AUD patients. Firstly, we used binarized and weighted DC methods to analyze the differences of the nodes in brain network between the two groups. The AUD group had significantly different binarized and weighted DC values in the left PreCG and the left IPL compared to HC group. Then, changes in the seed-based FC were used to further validate the results of DC. FC analyses indicated that AUD groups showed a decreased left PreCG/IPL-based FC in the right ITG, MTG, IOG, SPG, cerebellum_Crus 1, the left SFGdor, PCUN, LING and bilateral IPL. In this study, PreCG and IPL might be closely related to the occurrence of AUD. Through the statistical examination of relevant data, we found a statistically significant association between altered FC and clinical symptoms. Particularly, the FC values of the right MTG - right PreCG as well as the right IPL - left IPL were linked with MAST and ADS, respectively. This suggests that alternations of FC might be associated with AUD.

The AUD group had significantly different DC and FC values in the PreCG and the IPL. The PreCG region is known as the primary motor center of the brain [18], involved in the integration of information related to the sensory, motor, attention, and reward circuits [19–22]. Our findings of reduced DC and FC in the left PreCG region of AUD patients further enriched the evidence that chronic alcohol consumption might cause functional changes in the PreCG, which would affect sensory, motor and attention functions as well as brain reward mechanisms. And IPL is involved in attention allocation and assessment of decisions [23, 24]. Results from the present study suggest that patients with AUD have lower FC in a network involved in attention, which may account for the inability of AUD patients to make correct decisions and assessments. The cerebellum damage is also a risk with alcohol consumption. The most common central nervous system complication of AUD is cerebellar degeneration, which primarily manifests as cerebellar ataxia in approximately 10–25% of AUD [25]. Previous studies have reported extensive atrophy of the cerebellar gray matter in AUD patients [26]. AUD is associated with brain injury, particularly the frontal-parietal cerebellar gyrus, a circuit abnormality associated with changes in language, spatial working memory, executive function, gait and balance [27]. Alcohol-induced brain differences have also been observed in the right IPL with significantly higher amplitude of low frequency fluctuation (ALFF) values, conversely the ALFF values were lower in the posterior lobe of cerebellum and the left PCUN, suggesting disruption of the prefrontal-parietal-cerebellar circuit in individuals with AUD [28]. In this study, we found decreased functional connectivity in the left PreCG - left SFGdor, left IPL - right cerebellum_crus1/SPG, and left IPL - left PCUN in patients with AUD. This may indicate abnormal damage to the PreCG/IPL region, which affects the prefrontal-parietal-cerebellar circuit.

Reduced connectivity in the PCUN, LING, and IPL characterized the resting state functional network connectivity driven by drinking. These results are consistent with previous hypoconnectivity observations in visual and sensorimotor areas [29, 30]. Furthermore, as previously found, we identified the PCUN as a region associated with AUD [31]. The PCUN is thought to be engaged in visuospatial imagery, collection and evaluation of information, and self-processing operations [32–34]. Disturbed regional brain activity or functional connectivity in the PCUN was found in subjects with AUD and after heavy drinking [30, 35, 36]. Abnormal activation of the PCUN in subjects with AUD is thought to be associated with craving and visual memory processing [37]. The functional brain activity decrease in the PCUN may be interpreted as functional impairment caused by AUD. The significant FC differences were also observed in the right IOG and the left LING. These regions are involved in visual information processing and visual processing impairment is a common symptom in patients with AUD [38]. Previous research conducted by our team also revealed that impaired node efficiency was observed in visual cortex regions [10]. The visual dysfunction is prevalent in drinkers, however, the visual dysfunction associated to drinking is located around the LING, in contrast, the FC differences driven by both smoking and drinking are more frequently located about IOG [30]. Additionally, there is a correlation between activity in the visual cortex and vividness of mental imagery, suggesting that fMRI can detect individual differences in the vividness of mental imagery [39].

The AUD group exhibited a decrease in FC within SFGdor and SPG regions. The changes of these regions are consistent with previous studies and earlier work of our team [10, 40]. However, The ReHo study showed that SFGdor, PreCG, ITG and MTG are among the regions with stronger resting-state connections in individuals suffering from AUD [41]. Relapse after abstinence and reliance on alcohol can also be connected to aberrant frontal lobe neuronal activity in AUD patients, which may be related to failed abstinence behaviors in AUD patients [42, 43].

The regulation of the temporal lobe involves both vision and auditory senses [44]. The MTG is responsible for language-auditory processing, and the ITG is associated with the representation and detection of complex object features [45]. Long-term alcohol dependence is associated with compulsive drug-seeking behavior, which may stimulate the verbal, visual and auditory brain regions. Research by Bach et al. [46] demonstrated that patients with AUD exhibited a decrease in gray matter volume in bilateral MTG and ITG, which are primarily responsible for processing visual information and may be influenced by alcohol dehydrogenase genes. The interference of the brain reward network and ventral visual system with alcohol can result in affective changes and a decline in visual function [47]. The MTG, as part of the emotional circuit, plays a role in emotional responses. In an fMRI study on brain reactivity in response to emotional stimuli, the AUD group showed reduced responses to fear and disgust in the right amygdala, right MTG, pons, and left superior frontal gyrus, consistent with previous findings [48]. According to neurophysiological studies, the inferior temporal lobe played a role in the recognition of objects and the ventral stream of vision [49]. It is possible that the reduced connectivity in the right ITG correspondence correlates with impaired visual function, selectively affecting object discrimination and face recognition. In this study, weakened functional connectivity was found in the left PreCG - right ITG/MTG and left IPL - right ITG of AUD patients, potentially linked to impaired information communication and integration between corresponding brain regions, or abnormalities in the emotional circuit system. These findings may contribute to emotional instability, decreased attention, and changes in visual and auditory perception.

The dysconnectivity of the left PreCG - right MTG and the right IPL - left IPL might be possibly connected to clinical outcomes in AUD patients, as evidenced by the FC value of the left PreCG - right MTG being adversely correlated with MAST scores. Interestingly, we also discovered a positive correlation between the ADS scores and the FC value of the right IPL - left IPL, meaning that the AUD patients with lower FC values has a lighter dependence on alcohol. A previous study [11] showed that when patients with AUD had more severe cognitive impairment, as a compensation, they had less alcohol dependence. In conjunction with the current study, we can speculate that AUD patients with lower right IPL - left IPL FC values are less dependent on alcohol, which may be related to more severe cognitive impairment. There may be compensatory mechanisms between symptom severity and cognitive impairment, suggesting a difference in pathological pathways. These findings assisted future AUD studies by providing insight into possible mechanisms. Although we did not take the ADS score of HC group, HC has the higher FC values of right IPL-left IPL, which means the correlation between ADS and FC values of right IPL-left IPL cannot fit in HC group. These alterations in the mechanism between HC and AUD indicate an unseen functional role played by the right IPL-left IPL, which needs further explorations in future studies.

## Limitations

There were some limitations to be noted. Firstly, the sample size is relatively small, although rs-fMRI studies with more than 16 subjects per group are considered acceptable [50]. Secondly, this study is cross-sectional, which means we cannot directly link anomalous connections to AUD. Further longitudinal studies would be beneficial in addressing this issue. Thirdly, in our study, the cutoff for DC analysis was only set at r > 0.25 [36]. More thresholds should be used for comparison in future studies to reduce the effect of methodological choices on results. Fourthly, multiple analyses of psychometric-neural correlation data were not performed due to sample size, the purpose and methodology of this study.

## Conclusion

In summary, this study aimed to investigate the brain network of AUD patients using binarized DC, weighted DC and FC. The results showed extensive similarities in the spatial distribution of binarized and weighted DC values between the AUD and HC groups. Alterations in DC values in the brain network of AUD patients were found mainly in the PreCG and IPL. Besides, the FC within the in the right ITG, MTG, IOG, SPG, cerebellum_Crus 1, the left SFGdor, PCUN, LING and bilateral IPL were damaged in patients with AUD as well. Furthermore, the correlations between behavioral and node connectivity suggested that multiple inter-network connectivity dysfunctions played a compensatory role in AUD. DC and FC methods are valuable in detecting biomarkers / brain activity features specific for the AUD. These findings contribute to further understanding toward the brain network activities in patients with AUD.

## Data Availability

The datasets used or analyzed during the current study are available from the corresponding author on reasonable request.

## List of abbreviations

AUD: Alcohol use disorder
VBM: Voxel-based morphometry
DTI: Diffusion tensor imaging
WM: White matter
MRS: Magnetic Resonance Spectroscopy
rs-fMRI: Resting-state functional magnetic resonance imaging
DC: Degree centrality
FC: Functional connectivity
HC: Healthy controls
ADS: Alcohol drinking scale
MAST: Michigan Alcoholism Screening Test
MMSE: Mini-Mental State Examination
MoCA: Montreal Cognitive Assessment
TR: Repetition time
TE: Echo time
FOV: Field of view
FA: Flip angle
PreCG: Precentral gyrus
IPL: Inferior parietal lobule
MTG: Middle temporal gyrus
SFGdor: dorsolateral superior frontal gyrus
LING: Lingual gyrus
PCUN: Precuneus
SPG: Superior parietal gyrus
IOG: Inferior occipital gyrus
ALFF: Amplitude of low frequency fluctuation
MNI: Montreal Neurological Institute
BA: Brodmann area
L: Left
R: Right
